# Composite Membrane Dressings System with Metallic Nanoparticles as an Antibacterial Factor in Wound Healing

**DOI:** 10.3390/membranes12020215

**Published:** 2022-02-13

**Authors:** Angelika Kwiatkowska, Monika Drabik, Agata Lipko, Anna Grzeczkowicz, Radosław Stachowiak, Anna Marszalik, Ludomira H. Granicka

**Affiliations:** 1 Nalecz Institute of Biocybernetics and Biomedical Engineering, Polish Academy of Sciences, Trojdena 4 St., 02-109 Warsaw, Poland; ankwiatkowska@op.pl (A.K.); mdrabik@ibib.waw.pl (M.D.); alipko@ibib.waw.pl (A.L.); agrzeczkowicz@ibib.waw.pl (A.G.); 2 Institute of Microbiology, Faculty of Biology, University of Warsaw, Miecznikowa 1 St., 02-096 Warsaw, Poland; r.stachowiak@uw.edu.pl (R.S.); anna.marszalik@biol.uw.edu.pl (A.M.)

**Keywords:** wound healing, membrane dressings, bacteria, metallic nanoparticles as an antibacterial factor

## Abstract

Wound management is the burning problem of modern medicine, significantly burdening developed countries’ healthcare systems. In recent years, it has become clear that the achievements of nanotechnology have introduced a new quality in wound healing. The application of nanomaterials in wound dressing significantly improves their properties and promotes the healing of injuries. Therefore, this review paper presents the subjectively selected nanomaterials used in wound dressings, including the metallic nanoparticles (NPs), and refers to the aspects of their application as antimicrobial factors. The literature review was supplemented with the results of our team’s research on the elements of multifunctional new-generation dressings containing nanoparticles. The wound healing multiple molecular pathways, mediating cell types, and affecting agents are discussed herein. Moreover, the categorization of wound dressings is presented. Additionally, some materials and membrane constructs applied in wound dressings are described. Finally, bacterial participation in wound healing and the mechanism of the antibacterial function of nanoparticles are considered. Membranes involving NPs as the bacteriostatic factors for improving wound healing of skin and bones, including our experimental findings, are discussed in the paper. In addition, some studies of our team concerning the selected bacterial strains’ interaction with material involving different metallic NPs, such as AuNPs, AgNPs, Fe_3_O_4_NPs, and CuNPs, are presented. Furthermore, nanoparticles’ influence on selected eukaryotic cells is mentioned. The ideal, universal wound dressing still has not been obtained; thus, a new generation of products have been developed, represented by the nanocomposite materials with antibacterial, anti-inflammatory properties that can influence the wound-healing process.

## 1. Introduction

The clinical and economic burden of wound management increases globally every year. The trend is the most evident in developed countries with aging societies, where many patients suffer from civilization diseases. It is well known that comorbidities influence the effectiveness of the applied therapies, worsen injury healing, and lead to hardly curable chronic wounds. Despite significant efforts to reduce the risk of complications, the problem of difficult wound healing is still a major challenge for modern medicine. According to data gathered for the calendar year 2014 by Nussbaum and co-workers, based on the Medicare 5% Limited Data Set, total spending estimates ranged from USD 28.1 to USD 96.8 billion for all wound types in the United States [1]. On the other hand, The Guest Group estimates that the cost of wound care in the United Kingdom was at £4.5 to £5.1 billion in 2012 [2]. Generally, it is estimated that around 1.5–2 million people are suffering from both acute and chronic wounds across Europe [3]. It should be mentioned that prolonged therapies resulting from ineffective wound healing not only influences the budgets of national health institutions but also cause the deterioration in the quality of patients’ lives [3]. In order to reduce the economic burden on the system, several optimization programs have been introduced. For example, continuous improvement of health care procedures and training of medical personnel improves clinical practice and, thus, significantly lowers the therapy costs, providing benefits not only for patients and health care workers but also for the entire system [4]. On the other hand, a no less important aspect of wound healing therapy is optimal treatment using comprehensive dressings and therapeutics supporting the process [3]. Thus, it cannot be surprising that scientists worldwide have performed intensive studies on novel materials of specific parameters that could be potentially employed in wound dressings. It turns out that the achievements of nanotechnology may prove helpful. In recent years, it has become clear that nanomaterials have introduced a new quality in wound healing, opening the way to personalization and better adjustment of the dressing to the type of wound, environmental agents, or factors resulting from the patient’s burden with comorbidities. Furthermore, it was proven that the application of nanomaterials in wound dressing promotes injuries healing. Another important aspect connected to injuries care is bacteria participation, which becomes less clear-cut as knowledge of the wound healing process grows. To improve the effectiveness of wound therapy and consider the increasing multidrug resistance of bacteria, novel materials like nanoparticles are gaining popularity as antimicrobial factors. 

This paper presents the subjectively selected nanomaterials used in wound dressings, including the metallic nanoparticles, and refers to the aspects of their application as antimicrobial factors. The literature review was supplemented with the results of our team’s research on multifunctional new-generation dressings containing nanoparticles. The review discusses wound healing multiple molecular pathways, mediate cell types, and the affecting agents. Moreover, the categorization of wound dressings and some materials and membrane constructs applied in wound dressings are presented. The nanomaterials for wound dressings are presented, including the involvement of metallic nanoparticles. The mechanism of the antibacterial function of nanoparticles is discussed. Moreover, nanoparticles’ influence on selected eukaryotic cells is mentioned. Membranes involving NPs as the bacteriostatic factor for improving wound healing of skin and bones, including our experimental findings, are discussed. Some of our team’s studies concerning the selected bacterial strains’ interaction with material involving different metallic NPS, such as AuNPs, AgNPs, Fe_3_O_4_NPs, and CuNPs, are presented. Neither the ideal, universal wound dressing nor the perfect dressing material has yet been designed. Therefore, a new product generation of nanocomposite materials with antibacterial and anti-inflammatory properties supporting wound healing has been developed.

## 2. The Process of Wound Healing

### 2.1. Mechanism

Wound healing refers to a replacement of missing and devitalized cellular and tissue structures in living organisms [5]. Although the mechanism of the process is well known, discrepancies still appear in its characterization due to the complexity and dynamic course of its action. According to contemporary concepts, the human wound healing process consists of the four main stages: hemostasis, inflammation, proliferation, and remodeling with scar tissue formation [6,7,8]. On the other hand, the previous models do not distinguish hemostasis but treat it as a part of the inflammatory phase [9,10,11]. Moreover, some authors used maturation or resolution names instead of the remodeling or granulation names or instead of proliferation when describing the distinct stages. 

**Figure 1:**
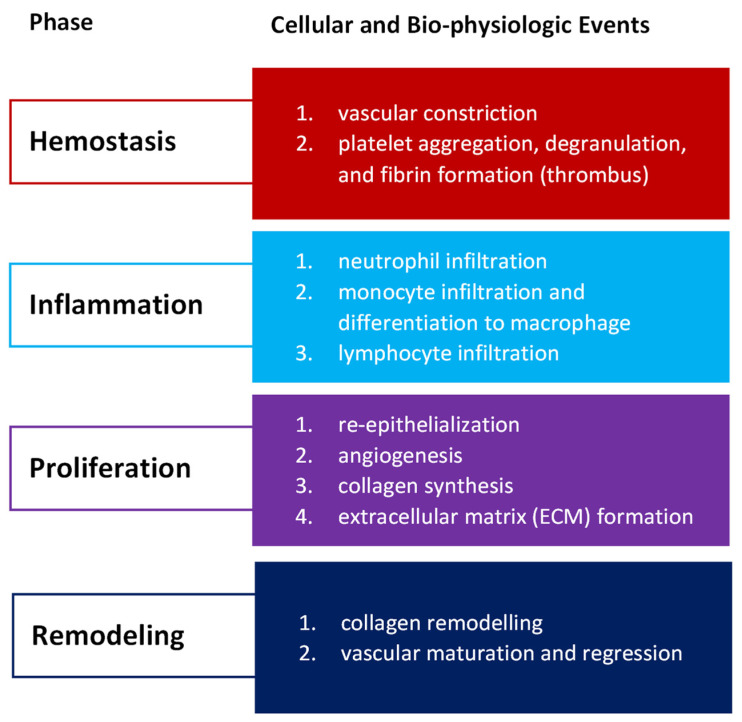
Cellular and bio-physiologic events occurring during the normal wound-healing process described by Gao and di Petro [12].

**Figure 2:**
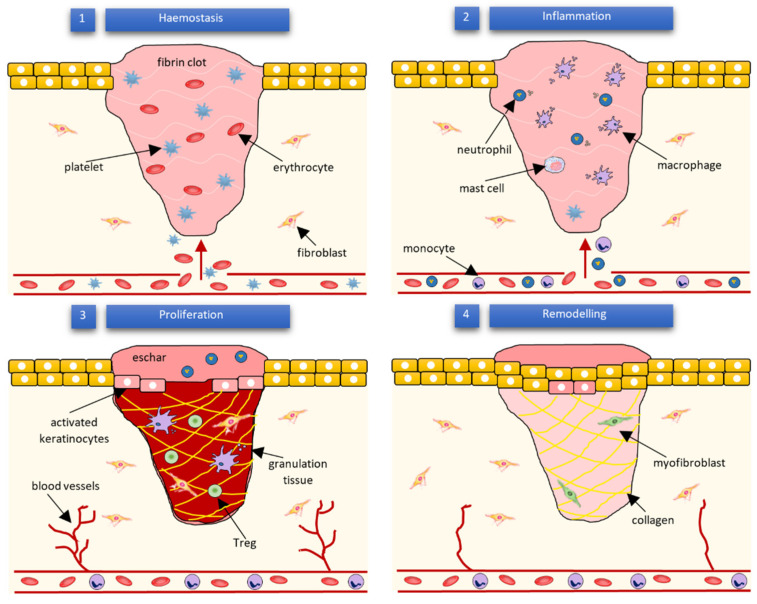
Subsequent phases of wound healing and their main cellular components according to Wilkinson and Hardman [13].

Wound healing success depends on the appropriate course of all successive stages. These phases have integrated with each other and occur in the specified time frames. Any aberrancies or interruptions disturb this highly programmed and precise process, resulting in complications and delays in the healing. In the worst case, chronic wounds might appear. During the hemostasis and inflammation stages, debris and bacteria are removed, exudates are coagulated, and blood is clotting. Simultaneously, the release of reactive oxygen species (ROS), proteases, growth factors (such as FGF—fibroblast growth factor, EGF—epidermal growth factor, TGF-β—transforming growth factor, and PDGF—platelet-derived growth factor [12]), and cytokines are triggered. Then, the epithelial cells migrate to the wound site (chemotaxis), and the proliferation phase can start, during which granulation tissue is formed, and angiogenesis and EMC secretion occur. Finally, wound contraction and reepithelization follow in the remodeling phase, resulting in scar tissue formation. As the appropriate environment must be formed, the whole wound healing process requires a long time to complete. The last stage can last from dozens of days (21 days) to one year. In Figure 1, we present the cellular and bio-physiologic events occurring during the normal wound-healing process, which Gao and di Petro described, whereas Figure 2 illustrates wound restoration stages together with their main cellular components.

Multiple molecular pathways and cell types mediate wound healing. Among other cells, the blood cells such as neutrophils, macrophages, and T-lymphocytes play a crucial role in the process. Neutrophils release ROS, and proteases as well are responsible for the removal of microorganisms, necrotic material, and cell debris occurring in the wound area [12]. On the other hand, macrophages produce cytokines [14,15], induce and clear apoptotic cells [16], and, after the phenotypic transition to reparative mode, promote the proliferation phase [17,18]. According to some authors, it is worth mentioning that efferocytosis (the process of apoptotic cells’ removal), which helps impede inflammation, might be a crucial function in the entire wound healing process [19]. The T-lymphocytes’ function is not yet completely understood. They are heavily involved in the inflammation [20] and re-epithelialization phases [6]. Nosbaum and co-workers proved that Foxp3-expressing regulatory T cells (Tregs) attenuate inflammation associated with injuries and facilitate cutaneous wound healing using the epidermal growth factor receptor (EGFR) pathway [21]. Other types of cells taking part in wound celling are fibroblasts, endothelial, and stem cells. The endothelial cells support the formulation and growth of new tissue [22]. For example, together with fibroblasts, they promote capillary growth [12]. Moreover, they are essential during hemostasis regulated by the dynamic balance between thrombocytes and endothelial cells, fibrinolysis, and coagulation [22,23]. On the other hand, fibroblasts support the collagen production and formulation of granulation tissue. Furthermore, they are responsible for producing the extracellular matrix components—glycosaminoglycans and proteoglycans [12]. Finally, it is considered that the contractile fibroblasts, termed myofibroblasts, mediate wound physical contraction [9,24]. Stem cells (SC) enhance wound healing by releasing growth factors and through paracrine signaling. They also influence the regeneration of damaged tissues [10,25]. Especially, adult stem cells like bone-marrow (BM)-derived cells (BMDCs) and epidermal stem cells have recently gained researcher attention. Epidermal stems give rise to the keratinocytes, which then re-epithelialize the wound [12]. Mesenchymal SC (MSC), present in the bone marrow, can differentiate into several cell types, among which fibroblasts, keratinocytes, osteoblasts, chondrocytes, and adipocytes can be enumerated [26]. On the contrary, hematopoietic SC (HSC), also found in bone marrow, gives rise to endothelial progenitor cells (EPCs) that are involved in neovascularization [27]. The presented list of cells contributing to wound healing is not complete—several other cell types are also involved—but it clearly shows the complexity of the process.

### 2.2. Factors Affecting Wound Healing

**Figure 3:**
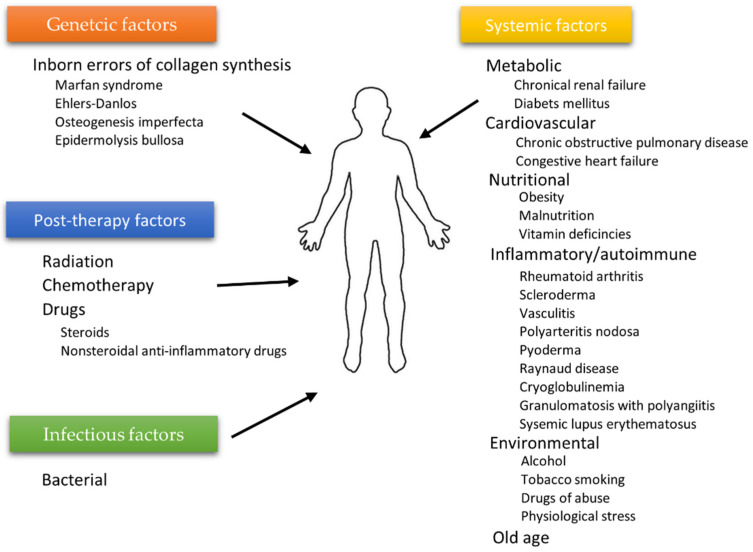
Factors affecting wound healing according to Beyene et al. [32].

The wound healing process depends on many individual and environmental factors. For example, the patient’s age weakening the body’s innate ability to regenerate [28], ischemia [29,30], or microbial infections often limit therapy’s effectiveness. Generally, two types of agents affecting wound healing can be highlighted: local factors directly influencing the wound characteristics and systemic ones related to the patient’s overall state affecting the healing process. Age, gender, stress, ischemia, chronic diseases, level of sex hormones, nutrition, underlying comorbidities, or addictions (alcoholism) are examples of factors from the second category, whereas infections, venous sufficiency, and oxygenation [31] can be categorized as local agents [12]. Some authors distinguish additional subcategories. The diagram in Figure 3 shows the division inspired by the work of the Beyene group [32].

Usually, a few factors are present simultaneously, leading to the increased impairment of wound healing. For that reason, the elimination of some of them can significantly improve the quality of treatment. Modern medicine applies many different techniques, reducing the action of unfavorable local and systemic factors. For example, hyperbaric oxygen therapy (HBOT) is applied to decrease the influence of chronic tissue hypoxia. Unfortunately, even though this method is adequate, it is not commonly available [33]. On the other hand, by using simple methods, the influence of inadequate nutrition, which worsens the ability to heal wounds, can be easily leveled [12]. Similarly, the bacterial infection risk can be overcome if the appropriate wound care is applied. Nonetheless, some factors affecting wound healing still represent a significant challenge in therapy. Especially, the agents related to the civilization diseases are problematic, as it is difficult to immediately improve the overall condition of the organism, which has been weakened by years of neglect [34,35]. 

More and more authors underline the significance of nutrition in the process of appropriate wound healing. Deficiencies of nutrients and malnutrition can reduce the ability to heal injures [12,36]. For that reason, contemporary treatment is becoming more holistic and includes the administration of therapeutics that not only work locally but also support the functioning of the entire organism. For example, arginine supplementation applied as an adjuvant treatment in wound care can achieve great results, as the metabolic demand for this amino acid increases under serving stress [24]. Arginine is indispensable during injury and periods of intensified growth because it influences the immune system and stimulates wound healing [37]. Similarly, it has been demonstrated that oral glutamine supplementation can increase the level of mature collagen and reduce the tendency of wounds to break [38]. On the other hand, achieving and maintaining the appropriate glucose level is crucial for diabetic patients’ therapy, as Arnold and Barbul reported [36]. Finally, the significance of protein doses in wound healing cannot be overlooked either. Protein–calorie malnutrition decreases T-cell function, drops phagocytic activity, depreciates antibody levels, and decreases wound tensile strength. As a result, the body loses its ability to protect the wound against infection [36]. The list of elements influencing healing is much longer. Micronutrients, vitamins, or fatty acids need to be present in sufficient amounts to facilitate the process [36]. In addition, the deficiency of individual elements might affect the healing pathways [12]. The supporting effect of proper nutrition during the therapy was shown perfectly in the Hayman group studies. The team’s clinical research indicated that the inclusion of high-energy supplements enriched by nutrition improved the healing of the pressure ulcer [39]. The next factor affecting wound healing is physiological stress. Studies have shown that stress significantly delays the process of healing by impairing the regular immunity at the injury site [40,41] Furthermore, it might cause unhealthy behaviors on psychological grounds (starting from anxiety and depression and ending with unfavorable habits such as alcoholism and abuse of drugs) that can be, on their own, detrimental to the wound healing process [12,42]. Advancing age is a known risk agent in reducing wound healing effectiveness [43]. This factor should be considered carefully, especially since the elderly population (over 60 years old) enlarges globally [12,43]. In recent decades, significant progress has been made in molecular and cellular biology, which has allowed scientists to broaden the knowledge of cell function loss with age. Many studies point to the impaired stress response of senescent cells as the leading cause of their dysfunction [28,30]. Moreover, several therapies reducing age-related wound healing impairment have been tested. For example, studies have shown that exercise can support cutaneous wound healing by stimulating anti-inflammatory response, decreasing the levels of pro-inflammatory cytokines in the injured tissues [44]. Nonetheless, with today’s level of development in medicine, it is not yet possible to eliminate the factors associated with the natural aging of cells. On the contrary, it is an entirely different matter for limiting the possibility of wound infections. The literature shows clinical examples of chronic wounds closed with success by lowering the bacterial count. Among others, perirectal wounds arising from fistulae or abscesses, pin tracts, and injuries with significant depth/surface area ratio were ultimately healed by applying this approach [28,45]. 

### 2.3. Bacteria Participation in Wound Healing

It should be noted that not all bacteria contribute to the wound healing process equally. Pathogens impede curative therapy [46], whereas commensal skin microorganisms positively affect treatment by regulating the innate immune response [47,48]. Modern studies indicate that the presence of bacteria promotes wound healing. Microorganisms can produce proteolytic enzymes, influencing the proteases’ release from neutrophils and supporting wound debridement [49]. However, for wounds that reach critical levels of bacterial colonization, the healing capacity decreases significantly, which is related to the metabolic load imposed by microorganisms [50]. Undoubtedly, skin microbiota composition analysis and monitoring are critical for developing novel treatment methods/wound dressings [51,52]. The excess of bacteria needs to be reduced to improve the effectiveness of chronic and acute wound therapy, but the balance of skin microbiota should be preserved simultaneously. For these reasons, and considering the increasing antibiotic resistance of bacteria, novel materials such as nanoparticles gain popularity as factors playing antimicrobial roles.

## 3. Wound Care Management 

**Figure 4:**
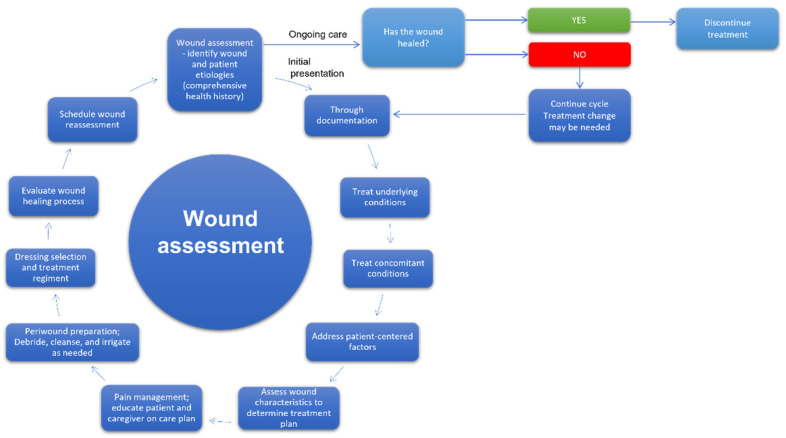
The cycle of wound assessment and decision on procedures [53,58].

The development of effective wound treatment is one of the main goals of modern medicine. According to Gupta and co-workers, wound management is a holistic cycle, encompassing the procedures related to the care of patients with wounds, starting from diagnosis and ending in follow-up. The steps of that ongoing process include wound recognition and characterization, risk assessment, selection of the optimal treatment, preparation of wound bed, monitoring, and taking practical actions depending on the observation results and evaluation of the effectiveness of the applied therapy [53]. Furthermore, effective wound care management can shorten the treatment and lower its costs, particularly in the case of chronic wounds therapy [54]. Therefore, medical personnel must pay special attention to adapting existing protocols to the individual needs of specific patients [55,56]. The appropriate dressing selection fitting the wound characteristic manifests such an approach [54,57]. In Figure 4, the model of a cycle of wound assessment and decision-making as to procedures is shown.

### 3.1. Wound Dressings 

The ideal, universal wound dressing does not exist. Some materials can be excellent for one type of injury, but that does not mean they will work for others. Each time, factors such as the profoundness of the wound or the exudates amount need to be considered before deciding on the applied dressing [59]. It is worth mentioning that the optimal wound dressing should be biocompatible, easy to apply, cheap, and, often, hypoallergenic [11,54]. Furthermore, it has to maintain a moist environment [60,61], ensure gas permeability [62], and simultaneously enable the extract of excess exudate from the injury area [59]. Furthermore, it ought to balance skin microbiota while protecting against infections [63]. The long list of the necessary wound dressing requirements generates the need for continuous research on the novel materials used in modern systems for injury healing.

Considering the wide range of wound dressing applications, it cannot be surprising that different classifications are used to categorize them [7]. First of all, taking into account the usage time, we can distinguish primary (directly applied to the wound) and secondary (employed to cover primary ones) dressings [64,65]. The second category relates to the interaction with the biological material (tissue) and embraces inert (passive) and bioactive (interactive) dressings. According to Weller and Sussman, passive dressings might be subclassified into absorbing and non-absorbing, whereas interactive dressings are grouped as absorbing, non-absorbing, and moisture donating [65]. Passive dressings have limited usage due to the permeability to bacteria and the high risk of adhesion to the wound. However, they are successfully applied for minimal and low-exudating wounds (like minor burns and simple, clean superficial wounds), mainly as a secondary dressing. They can also serve as primary dressings over skin grafts [65]. Moreover, the modern generation of passive dressings is characterized by better properties than their predecessors. For example, gauze dressing based on tulle and paraffin provides a waterproof layer protecting the injury. However, it should be noted that this type of cover is still not permeable for vapor and exudation; thus, it might cause maceration with prolonged usage [66]. Conversely, technologically advanced bioactive dressings interact with the wound to facilitate healing. Their main advantage is to provide moisture conditions in the wound area using the environment ensured by the organism. Bioactive dressings contain biologically active agents supporting wound healing, not rarely of antimicrobial function (e.g., growth factors, insulin, antibacterial compounds). They are made of different materials, including synthetic and natural polymers (e.g., elastin, hyaluronic acid, collagen, alginate), playing the role of active compounds or supporting biologically active factors [67]. The first line of bioactive dressings includes plasters immediately available for subacute and acute wounds, commonly used in clinical practice; the second line refers to unconventional (e.g., antimicrobial) dressings employed for complex chronic wounds. Among the examples of the first group are alginate [68,69,70], hydrogel [71,72,73,74], semi-permeable film [65], hydrocolloid [75,76,77], foam [78,79,80], and hydroactive [81,82] dressings [65]. In the second category, interactive hydrofibre [83], wet [65], silicone [84,85], honey [86,87,88], capillary wicking [89], hypertonic saline [90], silver [91,92,93,94], cadexomer iodine [95,96] dressings, and zinc paste bandages [97,98,99] are placed.

The last division references the material and technology applied to produce dressings; traditional, biomaterial-based, and artificial dressings are distinguished. Traditional plasters and bandages are based on cotton and gauze elements. They are treated as classic dressings, as they were used for centuries. Traditional dressings usually do not ensure appropriate moisture and must be changed frequently [57,100]. The biomaterial-based category includes allografts, xenografts, and tissue-engineered derivatives. This group includes skin substitutes, facilitating wound healing and closure of wounds by replacing skin function [101,102,103,104,105,106]. On the other hand, membrane, gel, spray, composites, foam, and film-based dressings are examples of the third type. 

### 3.2. Modern Wound Dressing Materials and Nanomaterials

It is impossible to fulfill the increasingly stringent wound dressing requirements (e.g., biodegradability, biocompatibility, and bioabsorbability) based only on classically used materials like rayon, woven, and non-woven fibers of cotton, polyesters, or rubber. Therefore, it cannot be surprising that different materials of unique properties and forms are applied in modern dressings. Especially polymers, of both synthetic and natural origin, garner great attention in the field; among the naturally occurring polymers employed in dressing production, chitosan [107], hyaluronic acid [108], keratin [109], gelatin, starch [110], silk fibroin [111], heparin [112,113], collagen [114], sericin [115], sodium alginate [116,117], zein [118], and konjac glucomannan [119,120,121] can be enumerated [122,123]. On the other hand, polyvinyl alcohol (PVA) [110], polylactic acid (PLA) [124], polyacrylic acid (PAA) [110], polyethylene glycol (PEG) [125], polycaprolactone (PCL) [126], and polyvinylpyrrolidone (PVP) [127] are examples of synthetic polymers applied for that purpose [128].

One of the most popular materials used in wound dressings is collagen. The role of this protein in wound healing is well described. The clotting cascade, resulting in a fibrin clot that stops the initial bleeding, is activated due to collagen exposure during the injury [129]. The mechanism by which coagulation allows for hemostasis in four phases of wound healing [130] is complex. The process occurs through a series of clotting factors using the extrinsic pathway (activated through tissue factor released by endothelial cells after external damage) and the intrinsic pathway activated through exposed endothelial collagen [131]. In the inflammation phase, Collagen I and IV fragments can be mediators of inflammation by acting as potent chemoattractants for neutrophils, enhancing phagocytosis and immune responses and modulating gene expression [129]. Inflammation is a critical step in the normal wound healing process and drives the proliferation of fibroblasts, which synthesize collagen and extracellular matrix (ECM) [129,132]. In 2019, the global collagen dressings market was valued at approximately USD 926 million [133,134]. Bovine origin collagen dominates the markets, and its composites with antimicrobial function have been the main subject of interest [133,134]. Many formulations of collagen dressings in the form of powder, gels, and films have been designed [135,136,137,138,139]. Another popular dressing material is chitosan, which might serve as the potential drug carrier because of its good biodegradability and biocompatibility and the potential to be modified by various chemical modifications to obtain desired properties. In addition, chitosan has a film-forming ability, and it is characterized by low immunogenicity. Finally, it has outstanding antibacterial and antifungal properties [140,141]. The latest example of chitosan application in wound dressing might be the 3D printed chitosan scaffolds proposed by the Hafezi group [142]. In turn, another material, nanoscale hyaluronic acid (HA), can improve cell adhesion on bone biomaterials and can provide significant mechanical reinforcement. It is one of the many biopolymers applied to prepare wound dressings, exhibiting several beneficial effects such as the decline in inflammatory processes and regulation of tissue remodeling [143,144]. It can be noted that the materials can be combined with each other and/or factors to ensure the expected properties. For example, the hydrogel formulation based on an antimicrobial peptide (AMP), epsilon-poly-l-lysine (EPL), and catechol, which was crosslinked via mussel-inspired chemistry between the amine and phenol groups, was reported. In vitro studies showed that EPL-catechol hydrogels possess remarkable antimicrobial and antibiofilm properties toward multidrug-resistant *A. baumannii* after three days of culture. In addition, a cytotoxicity study with the clonal mouse myoblast cell line (C2C12) revealed the good biocompatibility of this hydrogel [145]. Moreover, a sponge material prepared from a solution containing chitosan–polyvinyl alcohol emulsion with added polyhexamethylene guanidine hydrochloride in a homogeneous medium using lyophilization technology was explored. The applied material was reported to exhibit antibacterial function against multidrug-resistant *A. Baumanni* after 24 h, among others, allowing it to obtain an optical density significantly lower for the experimental group than for the control (untreated material). However, the visualization of bacterial cells within the bandage material was not presented [146]. Nevertheless, among the ready-made dressings on the market, the two hydrogels are mainly applied: alginate and collagen. In dressings based on collagen, their degradation rate and mechanical properties can be influenced by electrostatic cross-linking with chitosan or stabilization by hydrogen bonding with sugars or polyphenols [147]. Table 1 presents ready-made dressings using alginate and collagen as the primary substrates. 

The intensive development of nanotechnology observed in recent decades opened up an entirely new perspective for the field of wound dressings. Currently, nanostructured systems (materials with at least one external dimension measuring between 1 and 100 nm [148]) of various types are commonly applied as a part of the dressing to support wound healing. Among the different nanostructures used in the dressings, nanocolloids, nanocapsules, nanospheres, nanoemulsions, and nanoparticles can be listed [11]. 

Nanocolloids are composed of discrete entities of compounds (of the size of 1 to 100 nm) highly suspended/dispersed within a fluid medium (e.g., demineralized water). Nanocolloids occur in particulate form, wherein these particles might be organic or inorganic (e.g., non-ionic metal nanoparticles) in the crystalline or amorphous state. Nonetheless, the entities might also be formed by non-covalent molecular aggregates [156]. In addition, scattered particles may exhibit collective behavior. The particles are usually positively charged and have excellent electrical conductivity. In wound dressings, nanocolloids are used as antimicrobial agents, as they can penetrate eukaryotic and prokaryotic cells due to the perpetual Brownian motion [11]. However, some authors point to their potential cytotoxicity, limiting their usage on a mass scale [157]. 

Nanocapsules (NEs) consists of an oily or aqueous core surrounded by a distinctive polymeric membrane [158]. Their key advantage is the capability of encapsulation of active factors within their structure and their gradual release in time, which provides the controlled delivery. In addition, nanocapsules, applied as an element of the wound dressing, might increase the penetration level of active factors into deeper dermis layers, wherein the effectiveness of the compounds can decrease [159]. For example, an exciting example of the application of nanocapsules in wound healing therapy was reported by Guartinello and co-workers. The authors developed an antibacterial wound dressing composed of pH-responsive human serum albumin/silk fibroin nanocapsules immobilized onto cotton/polyethylene terephthalate (PET) blends loaded with eugenol [160].

Nanospheres (NSs) are tiny, uniform spherical systems built of a fixed porous biodegradable or nonbiodegradable polymeric core onto which biologically active agents (drugs, amino acids, plasmid DNA) can bind [161,162,163]. Thanks to this structure, active substances can be evenly distributed throughout the core, which improves their stability, biocompatibility, and general pharmaceutical properties and provides the possibility of the sustained, controlled release of loaded compounds [11]. Among the polymers, the one most often used to compose nanospheres, chitosan, polylactic acid (PLA), gelatin, and polylactide/glycolide might be mentioned [161]. Active factors are released from the nanosphere by diffusion, showing excellent drug-release profiles, especially for water-soluble agents [164]. The release time depends on the composition of the polymeric matrix, its loading capacity, and nanosphere size. Moreover, environmental factors like polymer core erosion, enzymatic degradation, or hydrolysis, causing the cleavage of polymer bounds and diffusion of the physically captured active substances, also influence the efficiency of the release process [164]. NSs are usually applied to entrapped hydrophilic or hydrophobic drugs [162,163]. The route of administration is compound-depended. For each substance, its intravenous targeting effect and controlled-release effects as well as its subcutaneous or intramuscular sustained-release, needs to be considered [161,165]. An example of the usage of nanospheres in wound dressing is ZnO-loaded chitosan/poly(vinyl alcohol)/acacia gum nanosphere-based nanocomposite thin films obtained through electrospraying [166]. Another interesting concept of dressing combining antimicrobial and regenerative properties was proposed by Müller et al. The authors designed three-dimensional (3D) electrospun poly(D,L-lactide) (PLA) fibermats into which nanospheres, formed from amorphous calcium polyphosphate (polyP) nanoparticles (NP) and encapsulated retinol (‘retinol/aCa-polyP-NS’nanospheres (NS)), had been incorporated [167].

Nanoemulsions (NEs) are homogenous, colloidal particulate forms in the submicron size range (droplet size of maximum 1000 nm; usually between 100 and 500 nm [168]) acting as carriers of drug molecules [159]. They are typically thermodynamically metastable oil-in-water (o/w) systems composed of emulsifier-coated oil droplets dispersed within an aqueous medium used to deliver hydrophobic active substances [169]. However, the multiple emulsions systems can be designed. Another example is water-in-oil (w/o) emulsions, where water droplets are dispersed in oil. These systems are applied for the delivery of hydrophilic compounds. More complex arrangements are water-in-oil-in-water (w/o/w) and oil-in-water-in-oil (o/w/o) emulsions [170]. Nanoemulsions can be formulated in many different forms, such as sprays, creams, liquids, and foams [171]. They show good fluidity and decreased viscosity [172]. Furthermore, the small-sized droplets have a greater surface area, providing better absorption of active agents [171]. According to Aswathanarayan and co-workers, NEs, in contrast to microemulsions, are not affected by physical and chemical variations, including temperature and pH [170] a completely different view than that presented by Chime Group [168]. Insufficient comprehension of the mechanisms of the biological fate of nanoparticles after digestion and the potential toxicity of engineered nanoemulsions limit their application in the food industry and medicine [170]. Additionally, the next great challenge connected with the popularisation of NEs’ usage is the high costs of their production, requiring investments in special equipment (e.g., homogenizers) [168]. Nevertheless, the NEs have still been explored, e.g., nanoemulsion of clove oil was reported to present significant wound healing effects in rats compared to pure clove oil [173].

Nanoparticles (NPs) used in wound treatment can be divided into two groups: nanoparticles served as delivery systems for active compounds and nanoparticles of specific intrinsic properties supporting injuries healing [11]. These are composed of metallic (silver, gold, copper, and zinc NPs), metal oxide (copper oxides, ferroxides), or nonmetallic (fullerenes and carbon NPs, polymer nanoparticle (PNP), lipid-based NPs, ceramic NPs) nanomaterials. 

Metal nanoparticles such as silver, gold, and zinc are usually integrated into a wound dressing to provide antimicrobial properties and facilitate wound healing. Due to their unique structure, they are characterized by the increased surface-to-volume ratio, which lowers their concentration in the dressing [159]. Among the factors influencing the biological behavior of nanoparticles, their chemical structure and heterogeneity, porosity, and hydrolytic stability can be enumerated. The abovementioned characteristics determine how the nanoparticles interact with other biomolecules, and thus indirectly affect their biodistribution [11]. 

Generally, it is assumed that nanoparticles exhibit low cytotoxicity [11]. However, some scientists raise the issue of the mechanism of nanoparticles accumulation in live organisms, which is not well studied yet [174,175,176,177] especially, that nanoparticle toxicity is related to their structure (dimension and architecture) and characteristics (particle surface charge described by zeta potential, value of polydispersity index, surface functionalization) [178]. Most often, the smaller the particles, the greater their biological activity. Similarly, the particle surface charge determines its capability for penetrating cellular barriers and its capacity for receptor binding [179]. According to Auffan and co-workers, the potential cellular toxicity of nanoparticles depends on their chemical stability [180].

Nanomaterials employed in wound dressings can occur in the form of individual nanoparticles or as nanocomposites. Moreover, coatings and scaffolds are also often used. 

There are two main approaches for nanomaterial usage in wound healing therapies: (1) nanomaterials of necessary properties are incorporated into polymers, or (2) nanocarriers are applied for the encapsulation of the other materials, mainly active agents [181]. Nanoelements are embedded in support materials to improve their antimicrobial properties (metallic nanoparticles), stiffen the dressing structure (e.g., fullerenol), mimic the extracellular structure (nanoscafolds), or support cell growth (nanofibers). As a result, the designed dressing materials can be customized and adjusted to the specific wound types. On the other hand, the possibility of closing the active agent in nanoelements provides the opportunity for targeted delivery of biologically active substances (growth factors, drug). Barroso and co-workers have collected the most popular therapeutics (bioactive agents, drugs, oligonucleotides, nitric oxide, and plasmid DNA) encapsulated within the various types of nano-sized carriers. The division which they proposed is shown in Table 2. 

Another critical aspect of nanomaterial usage in wound healing is to fit the applied material to the appropriate phase of the process. For example, gold nanoparticles usually serve as an anti-inflammatory agent; however, the studies show that AuNPs also support epidermal re-epithelization through the proliferation of the keratinocytes. In turn, carbon nanotubes properties are used during inflammation, whereas iron oxide NPs are most effective during proliferation and remodeling stages. Therefore, the developed dressing should be constructed to emphasize the advantages of different materials. For example, the nanoelements needed in the later phases of wound healing should be placed in the inner layers of the multilayer dressing. Various therapeutics delivered by nanocarriers in wound healing therapies are presented in Table 3.

To summarize, many different nano-based systems were designed and tested to be used in wound therapies, especially as a part of modern wound dressings. Each of them has various properties and might be applied to achieve specific characteristics of the resulting material. For example, nanofibers can replace artificial dermal analogs, as they provide favorable conditions for cell–drug integration and cell attachment. In contrast, nanoscaffolds imitate the properties of the extracellular matrix by mimicking the fibrous nature and nanoscale features of skin elements. In turn, inorganic particles serve as antibacterial agents and support wound healing. On the other hand, polymeric particles’ properties are beneficial in the controlled release of active agents and protect the drug from degradation by wound proteases. Finally, liposomes ensure a moist environment on the wound surface and provide sustained drug release. Similarly, other lipid particles (like nanostructured lipid carriers or solid lipid NPs) favor the controlled release of active factors and secure the administration’s versatility [181]. The final decision on applying a nano-based system depends on the wound-healing phase, the expected therapeutic effect, injury characteristics, and drug parameters (required dose, mechanism of action).

## 4. Nanoparticles and Mechanism of Antibacterial Function 

The broad spectrum of antibacterial activity of the nanoparticles makes them an inherent element of biomaterials. For example, using the NPs in modification of tantalum oxide coatings by attaching silver nanoparticles (2.61% concentration), it was found that AgNPs improve material antibacterial properties against *Staphylococcus epidermidis* while maintaining the function of (rat bone mesenchymal stem cells) tested in the system [182]. In addition, modification of polyvinyl chloride endotracheal tubes with gold nanoparticles shows bacteriostatic activity against *Listeria monocytogenes* [183]. Another example of the usage of silver nanoparticles is an attempt to reduce complications related to the application of intravenous ports. However, it should be noted that [184] clinical trials comparing polyurethane AgTive^®^ silver-impregnated or unmodified central intravenous catheters found that silver-impregnated catheters did not reduce the incidence of catheter-related infections spreading downstream of the bloodstream compared to standard intravenous central catheters. This theory is also confirmed by the meta-analysis conducted in 2014 by Chen et al. [185]. 

The mechanism of the antibacterial action of soft transition metal nanoparticles, including silver and gold, is not well understood. Some researchers suggest that the antibacterial activity of silver nanoparticles may be due to the damage they cause to the cell membrane. They observed that the accumulation of AgNPs (5–10 nm) on the cell wall of *Escherichia coli* leads to perforation, which results in the loss of integrity of the bacterial outer membrane. In addition, biomolecular and structural damage has been reported in lipopolysaccharide and phosphatidylethanolamine [186]. NPs can penetrate the cytoplasm of the bacterial cell and damage intracellular structures [187]. It was noted that AuNPs could penetrate *Streptococcus pneumoniae* bacteria, generating the formation of a spherical cytoplasmic structure called the inclusion body of gold nanoparticles, the composition of which remains unknown [188]. Another mechanism of antimicrobial activity results from the oxidation of soft transition metal nanoparticles in contact with water, which leads to the release of ions [189]. The bacteriostatic effect of the released ions is closely related to their interaction with the thiol groups of enzymes and proteins associated with the cell wall, disrupting the respiratory chain and the function of the bacterial cell wall [190]. Inhibition of major proteins in the respiratory chain (e.g., cytochrome b) causes an increase in reactive oxygen species (ROS) such as hydrogen peroxide (H_2_O_2_), hydroxyl (OH–), and peroxide (O_2_–), radicals inside the cell, which leads to oxidative stress and protein and nucleic acids damage, with subsequent bacterial death [172,189,191,192,193,194]. The DNA damage includes nuclear fragmentation [195] or physical attachment of the NPs to the DNA [196]. In addition, the exposure of bacteria to NPs causes genomic changes such as gene upregulation, downregulation, and expression levels in dependence on bacterial strain and nature of NPs, e.g., AgNPs regulate 309 genes in *E. coli* [197]. Subsequent studies show that affecting *P. melaninogenica* and A. *pyogenes* AgNPs is mainly related to oxidative and nitro-oxidative stress induced by ROS generation, which results in increased leakage of dehydrogenase and NO [198].

## 5. Nanoparticles’ Interaction with Eukaryotic Cells

Regarding the interaction of NPs with eukaryotic cells, reports present the assumption that NPs cross the cell membrane and mitochondria [175,199]. Similar to the mechanism of toxicity for bacterial cells, silver nanoparticles toxicity is connected to the overproduction of ROS affecting the respiratory chain of mitochondria, DNA damage [200], and apoptosis [201]. 

As far as skin penetration is concerned, it has been found, among others, on a rodent model that AuNPs of 15 nm diameter aggregate in deeper layers of the skin, while larger AuNPs (about 100–200 nm) reach only the epidermis and derma [202]. In turn, particles with an iron core with a diameter equal to or less than 20 nm can reach the living epidermis of human skin [203].

Moreover, there are reports that AgNPs coated with polyvinylpyrrolidone 10–50 nm in size showed, at TEM assessed penetration studies, that their presence in the stratum corneum and electrothermal atomic absorption spectroscopy allowed for their penetration into the water compartment [204]. Attention should be paid to the possibility of endocytosis of NPs by eukaryotic cells, which may be disadvantageous in dressings, although it is desirable for drug delivery systems functioning. There are some reports of NPs’ interaction with different eukaryotic cells, for example, lung cell lines A549 and 95D. 

It was found that AuNPs are endocytosed by lung tumor cells, and, additionally, the influence of AuNPs on the invasiveness of lung cancer cells in vitro was proven. AuNPs with a diameter of 5 nm can stop the growth of A549 cells (human lung cancer cell line) but at the same time increase the invasiveness of these cells. The AuNPs at 10 nm diameter increases the invasiveness of 95D cells (non-small cell lung cancer). Such effects are not observed for larger NPs with 20–40 nm dimensions. Increased invasiveness may be associated with increased expression of metalloproteinase-9 in the matrix and the intracellular adhesive molecule-1. This result suggests that metalloproteinase-9 and intracellular adhesion Protein-1, the key invasiveness modulators, are regulated by AuNPs [205]. Moreover, some authors examined the influence of CuO, Fe_3_O_4_, and Fe_2_O_3_ NPs on A549 cells. However, a high variation among nanoparticles concerning their ability to cause toxic effects was observed; CuO nanoparticles were most potent regarding cytotoxicity and DNA damage. No or low toxicity was observed for iron oxide particles (Fe_3_O_4_, Fe_2_O_3_) [206].

The assessment of the influence of copper oxide nanoparticles (CuONPs) on human laryngeal epithelial cells proved that induction of cytotoxic influence is associated with the increase in reactive oxygen species [207]. Moreover, it was demonstrated that the CuNPs showed higher toxicity than their oxide nanoparticles CuONPs in HL60 cells [208].

AgNPs are applied in orthopedic implants because of their antibacterial properties and possible enhancement of mineralization of osteoblast or osteoblast-like cells [209,210,211].

Nevertheless, some authors demonstrated that AgNPs < 100 nm inhibited the differentiation and mineralization of osteoblast-like cells MG-63 due to uptake and retention of AgNPs. Furthermore, cytotoxic effects of low-dose AgNPs on MG-63 cells persisted even after termination of exposure in a 72-h experiment [212]. 

Our team studied, on human bone cells hFOB and fibroblasts HDF, the internalization of AuNPs of about 10 nm diameter, which were part of nanocomposite elements of the dressing developed for cooperation with the bone–skin interface. However, the internalization of NPs occurred and no cytotoxic effect against the hFOB and HDF cells was observed. Moreover, it was found that the participation of fullerenol within the membrane layer stops the internalization of AuNPs by human osteoblasts hFOB [213]. 

Designing material for cooperation with eukaryotic cells is essential to balance bacteriostatic and cytotoxic activity.

**Figure 5:**
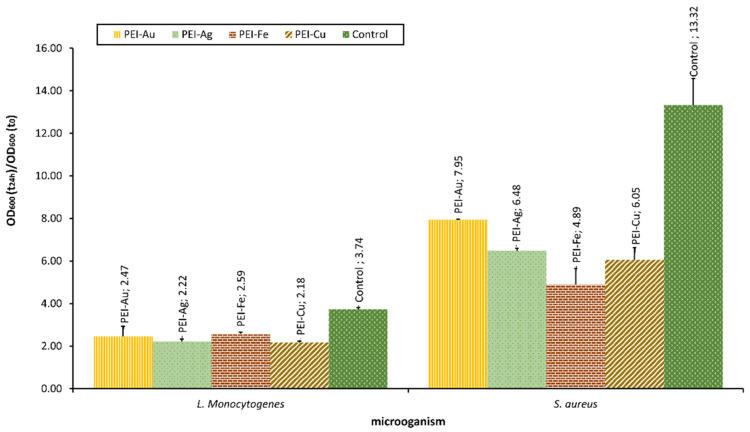
The optical density of bacterial strains L. monocytogenes and S. aureus after 24-h culture in the presence of nanocomposite membrane material based on polyethyleneimine (PEI) with incorporated metallic NPs. Key to the symbols: PEI-Au—polyethylenimine incorporating gold nanoparticles; PEI-Ag—polyethylenimine incorporating silver nanoparticles; PEI-Fe—polyethylenimine incorporating Fe_3_O_4_ nanoparticles; PEI-Cu—polyethylenimine incorporating copper nanoparticles. The values are presented as mean ± SD.

Our team examined the selected bacterial strains’ interaction with material involving different metallic NPS, such as AuNPs, AgNPs, Fe_3_O_4_NPs, and CuNPs. The optical density of bacterial strains after 24-h culture in the presence of membranes incorporating metallic NPs is presented in Figure 5.

The applied NPs proved to exert bacteriostatic influence towards both *L. monocytogenes* and *S.aureus* strains. Furthermore, there was a statistically significant difference between the OD of *L. monocytogenes* or *S. aureus* after 24 h of culture in the presence of material with incorporated metallic nanoparticles and the control. Material involving CuNPs proved a more substantial bacteriostatic effect on *L. monocytogenes* than the other membranes. This observation reflects the effect on eukaryotic cells observed by some authors, indicating the more decisive influence of CuNPs compared with other metallic NPs. In the case of *S. aureus*, this pattern was not observed. There was no statistical difference between the membranes involving CuNPs and Fe_3_O_4_NPs. 

## 6. Membranes Involving NPs as the Bacteriostatic Factor for Dressings for Wound Healing of Skin and Bones 

Such elements as metallic nanoparticles, such as Cu, Zn, and silver (Ag), can prevent infections via their antibacterial activity [214]. For example, wound dressings based on chitosan, such as chitosan/carboxymethyl chitosan/silver nanoparticles (CTS|CMCTS|AgNPs) polyelectrolyte composite based on natural polymers with no chemical reductant involved have been examined for this purpose. The application of CTS|CMCTS|AgNPs hydrogel to wound healing performed using the *P. aeruginosa* infected wound mice model demonstrated slightly better effects than FAC, the widely used cream in clinics [214]. Some authors explored nanosilver particles-collagen/chitosan hybrid scaffolds’ (NAg-CCS) performance in vivo on a rodent model, observing the improved condition of the wound bed and the progress of normal inflammatory stage without unwanted extension or aggravation, which would allow avoiding scar formation. Moreover, the minimal inhibitory concentration of AgNPs in the scaffold, observed on *S. aureus* and *E. coli*, was ≤10 ppm [215]. Another membrane fabricated of chitosan/nano- hydroxyapatite/nanosilver composites proved that the material was non-toxic to rat osteoprogenitor cells and human osteosarcoma cell line and exerted broad-spectrum antibacterial activity against Gram-negative and Gram-positive bacteria [216]. The composition of hydroxyapatite coatings with silver NPs was applied to find the balance between the optimal osseointegration and antimicrobial properties of coated commercially available TiAl6V4 alloy implants [217]. However, bacterial resistance to silver is low; during over 40 years, *E. coli*, *Enterobacter cloacae, Klebsiella pneumoniae, Acinetobacter baumannii, Salmonella typhimurium,* and *Pseudomonas stutzeri* have been reported as silver-resistant strains [218,219,220,221,222]. One of them, *A. baumanni,* a Gram-negative coccobacillus, was recently listed as the “number one” critical level priority pathogen because of the significant rise of resistance against antibiotics [223].

**Figure 6:**
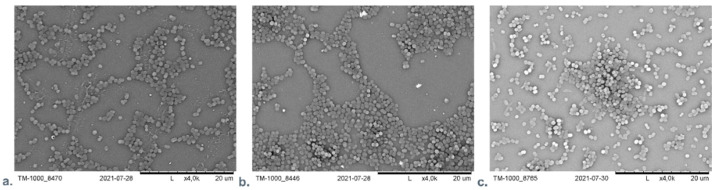
The SEM visualization of nanocomposite material based on polyethyleneimine (PEI) with incorporated AuNPs or AgNPs with *A. baumanni* after 24-h cultivation. (**a**): PEI—AuNPs membrane; (**b**): PEI—AgNPs membrane; (**c**): Control—*A. baumanni* after 24 h of cultivation without membrane.

Our team examined the difference of influence of AuNPs or AgNPs on *A. baumanni* after 24 h cultivation in the presence of material with incorporated nanoparticles. No significant difference was found between the AgNPs and the AuNPs containing material using SEM microscopic analysis. Figure 6 presents the SEM visualization of different nanocomposite materials with *A. baumanni.*

**Figure 7:**
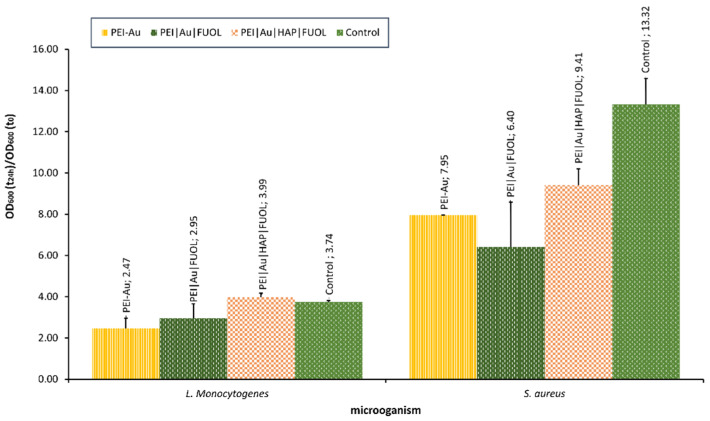
The optical density of bacterial strains *L. monocytogenes* and *S. aureus* after 24-h culture in the presence of nanocomposite membrane material based on polyethyleneimine (PEI) with incorporated AuNPs. Key to the symbols: PEI-Au—polyethylenimine incorporating gold nanoparticles; PEI|Au|FUOL—polyethylenimine incorporating gold nanoparticles and fullerenol, PEI-Au|HAP-FUOL—polyethylenimine incorporating gold nanoparticles and hydroxyapatite mixed with fullerenol. The values are presented as mean ± SD.

Our team is currently studying the influence of nanocomposite materials on selected bacterial cells. For example, the interaction with materials involving Au, constructed for bone–skin interface [213], was assessed. The materials proved to be non-toxic towards the human skin cells, and osteoblasts [213] exhibited some bacteriostatic influence. It was observed that the NPs involvement in polyethyleneimine allowed for OD decline compared with the control (bacterial strains cultivated without the membrane film presence) in *S. aureus*. In the case of *L. monocytogenes*, OD values did not significantly differ for examined membranes involved, except for Au, some other additives such as HAP and/or FUOL and control. The optical density of bacterial strains after the 24-h culture in the presence of membranes designed for the bone–skin interface is presented in Figure 7.

## 7. Conclusions

Nevertheless, there is still no superior substitute for patients’ tissues for reconstruction purposes; the field of wound care is constantly being explored in the name of developing new technology and products to facilitate healing. The new product generation consists of nanocomposite materials with antibacterial and anti-inflammatory properties that influence wound healing. Nevertheless, there are concerns regarding their usage in the clinic, such as possible adverse effects on eukaryotic cells. Thus, a balance between bacteriostatic and cytotoxic activity is vital in materials design. Moreover, the problem of bacterial resistance arises due to bacterial adaptation to silver, the mechanism of which is unexplored. Therefore, the design of the material ensuring antibacterial activity against microorganisms, including multidrug resistance, is a constant challenge. 

## Figures and Tables

**Table 1:** Materials based on collagen and alginate in the form of commercially available dressings.

Material	Commercially available dressings
**Collagen**	
collagen/chitosan collagen/chitosan/glycosaminoglycans collagen/glycosaminoglycan (chondroitin-6-sulphate) collagen/oxidized regenerated cellulose collagen/oxidized regenerated cellulose/AgNPs collagen/tricalcium phosphate β-Ca_3_(PO_4_)_2_	Kollakhit [149]
	Kollakhit-Bol [149]
	Integra (Integra Life Sciences) [150]
	Promogran (Systagenix Wound Management) [151]
	Promogran Prisma [152]
	Vitoss granules (Orthovita) [153,154]
**Alginate**	
alginate/AgNPs	Aquacel Ag^®^; Biatain^®^ Alginate Ag; CuraFoam™ AG Silver Foam Dressing; DynaGinate™ AG Silver Calcium Alginate Dressing; Dynarex^®^ DynaFoam™ AG Bordered Silver Foam Dressing [155]

**Table 2:** Various therapeutics delivered by nanocarriers in wound healing therapies, according to Barroso et al. [181]. Key to symbols: NPs—Nanoparticles.

Nanocarrier	Therapeutic				
	Bioactive Agent	Drug	Oligonucleo-tide	Nitric Oxide	Plasmid DNA
Ceramic NPs		x		x	
Dendrimers			x		
Gold NPs		x			
Iron oxide NPs				x	
Liposomes	x	x	x		
Micelles		x			
Polymeric NPs	x	x			x
Silver NPs		x	x		x
Solid Lipid NPs	x	x			

**Table 3:** Various therapeutics delivered by nanocarriers in wound healing therapies, according to Barroso et al. [181]. Key to symbols: NPs—Nanoparticles.

Nanocarrier	Phase			
	Hemostasis	Inflammation	Proliferation	Remodeling
Carbon nanotubes		x		
Ceramic NPs	x	x	x	
Copper NPs			x	
Dendrimers			x	
Gold NPs		x	x	
Iron oxide NPs			x	x
Liposomes		x	x	
Micelles			x	
Nanoceria NPs	x	x	x	x
Polymeric NPs	x	x	x	
Silver NPs		x	x	
Solid Lipid NPs			x	
